# The Safety and Efficacy of Peptide Receptor Radionuclide Therapy for Gastro-Entero-Pancreatic Neuroendocrine Tumors: A Single Center Experience

**DOI:** 10.3390/curroncol31090416

**Published:** 2024-09-18

**Authors:** Leandra Piscopo, Emilia Zampella, Fabio Volpe, Valeria Gaudieri, Carmela Nappi, Erica Di Donna, Stefania Clemente, Antonio Varallo, Mariano Scaglione, Alberto Cuocolo, Michele Klain

**Affiliations:** 1 Department of Advanced Biomedical Sciences, University of Naples, Federico II, 80131 Naples, Italy; emilia.zampella@gmail.com (E.Z.); fabio.volpe@unina.it (F.V.); valeria.gaudieri@unina.it (V.G.); c.nappi@unina.it (C.N.); erica.didonna@studentiunina.it (E.D.D.); edrclementeaou@gmail.com (S.C.); antonio.varallo@hotmail.it (A.V.); cuocolo@unina.it (A.C.); micheleklain@libero.it (M.K.); 2 Department of Medicine, Surgery and Pharmacy, University of Sassari, 07100 Sassari, Italy; mscaglione@uniss.it

**Keywords:** gastro-entero-pancreatic neuroendocrine tumors (GEP-NETs), peptide receptor radionuclide therapy (PRRT), [^177^Lu]Lu-DOTA-TATE, multidisciplinary team, dosimetry

## Abstract

The aim of the present study was to evaluate the safety and efficacy of radionuclide therapy with [^177^Lu]Lu-DOTA-TATE according to our single center experience at the University of Naples Federico II. For the present analysis, we considered 21 patients with progressive, advanced, well-differentiated G1 and G2 in patients with gastro-entero-pancreatic (GEP) neuroendocrine tumors (NETs) treated with [^177^Lu]Lu-DOTA-TATE according to the decisions of a multidisciplinary team. All patients underwent four cycles of 7–8 GBq of [^177^Lu]Lu-DOTA-TATE every 8 weeks. A whole-body scan (WBS) was performed 4, 48, and 168 h after each treatment. The dosimetry towards the organ at risk and target lesions was calculated. For each patient, renal and bone marrow parameters were evaluated before, during, and 3 months after the end of the treatment. Follow-up data were obtained and RECIST criteria were considered as the endpoint. Among 21 patients enrolled (mean age 65 ± 9 years); 17 (81%) were men and the small intestine was the most frequent location of disease (n = 12). A mild albeit significant variation (*p* < 0.05) in both platelets and white blood cell counts among all time points was observed, despite it disappearing 3 months after the end of the therapy. According to the RECIST criteria, 11 (55%) patients had a partial response to therapy and 8 (40%) had stable disease. Only one (5%) patient had disease progression 4 months after treatment. Our data confirm that [^177^Lu]Lu-DOTA is safe and effective in controlling the burden disease of G1/G2 GEP-NETs patients.

## 1. Introduction

The neuroendocrine tumors (NETs) are a heterogeneous group of neoplasms sharing the ability of overexpressing somatostatin receptors (SSTR), a well validated target for both diagnostic and therapeutic purposes. The introduction of peptide receptor radionuclide therapy (PRRT) using a theragnostic approach has demonstrated a significant impact on the management of NETs patients [1,2,3,4,5]. Theragnosis is a branch of nuclear medicine that combines diagnosis and therapy by using the same targeting agent [6,7,8]. In NET patients, this approach has been applied for both diagnostic and therapeutic purposes by using SSTR analogs that can be labeled with Gallium-68 (^68^Ga) for positron emission tomography/computed tomography (PET/CT) imaging, and with lutetium-177 for therapy [8]. Lutetium177 has the ability to emit β−radiation during nuclear decay, leading to tumoral cellular death through DNA damage, with a longer residence time in tumors and a lower kidney exposure as compared to other β−emitters [9,10]. The safety and efficacy of [^177^Lu]Lu-DOTA-TATE (Lutathera^®^) were first validated by the NETTER-1 trial, a pivotal phase III randomized protocol [2,3]. The 229 patients enrolled were randomized into two groups and assigned to receive high doses of octreotide alone or 7.4 GB of [^177^Lu]Lu-DOTA-TATE at 8 weeks intervals for four cycles, in association with octreotide. In the first interim analyses, the investigators observed a significantly higher PFS rate in the PRRT group (65.2%) vs octreotide group (10.8%). Moreover, the [^177^Lu]Lu-DOTA-TATE group had a higher rate of positive response to therapy versus the control group (18% vs. 3%). The results of the NETTER-1 trial confirmed the superiority of [^177^Lu]Lu-DOTA-TATE over high-doses of octreotide alone in patients with progressive, advanced, well-differentiated G1 and G2 gastro-entero-pancreatic (GEP)-NETs, opening the way to radiopharmaceutical registration. In Italy, PRRT using [^177^Lu]Lu-DOTA-TATE was introduced several years after the NETTER-1 results were published and radiopharmaceuticals have been approved in Europe. However, the feasibility of PRRT using somatostatin analogs requires adequate resources according to European standards, such as hospital personnel, standardized procedures, and both radioprotection and dosimetry devices. Moreover, the decision of addressing GEP-NETs patients on PRRT protocols should be taken by a multidisciplinary team, including several figures such as oncologists, endocrinologists, nuclear medicine physicians, and surgeons. For these purposes, the European Neuroendocrine Tumor Society (ENETS) has been founded as a multidisciplinary network that aims to improve both diagnostic and therapeutic pathways of NETs patients [11]. In this context, as part of the ENETS network at the University Federico II of Naples, we have been able to perform PRRT in GEP-NETs patients since 2020. It should be considered that, despite encouraging results from NETTER-1 in real clinical practice, several factors, including patients’ selection criteria and available resources, should be considered in the evaluation of safety and efficacy of PRRT in GEP-NETs. We aimed to utilize the results of our single center experience with [^177^Lu]Lu-DOTA-TATE therapy at the University Federico II of Naples to evaluate the safety and efficacy of PRRT in patients with GEP-NETs.

## 2. Materials and Methods

### 2.1. Patient Recruitment, Selection and Management

This is a 5-year retrospective observational study conducted in a population of patients with well-differentiated GEP-NET who were referred to PRRT with [^177^Lu]Lu-DOTA-TATE from October 2020 to November 2023 at the Department of Radionuclide Therapy, University Federico II of Naples, Italy. As already mentioned, according to the “ENETS” recommendations, in our region, all NET patients undergo multidisciplinary evaluation by several figures, including endocrinologists, oncologists, radiologists, and nuclear medicine physicians. Therefore, the therapeutic recommendation of the ENETS group is mandatory [11]. All patients were referred to [^177^Lu]Lu-DOTA-TATE treatment according to the following inclusion criteria: the presence of histologically proven GEP-NET with locally advanced and/or inoperable metastatic disease; a failed first-line systemic therapy; evidence of progressive disease while on SSA therapy or uncontrolled symptoms despite systemic therapy; SSTR expression on the known tumor lesions demonstrated by [^68^Ga]Ga-DOTA-peptides PET/CT scan within the past 6 months; and an ECOG status 0–2. Moreover, written informed consent was obtained for all patients. We excluded all the patients with histological evidence of grade 3 neuroendocrine carcinoma, severe acute concomitant illnesses or psychiatric disorder, a life expectancy < 3 months, poor renal function, defined as an effective glomerular filtration rate (eGFR) < 40 mL/min, and women during pregnancy or during breast feeding. Demographic and clinical data were obtained for each patient, as well as the results of laboratories and imaging procedures performed before and after each cycle of treatment.

### 2.2. Therapy and Imaging Protocol

All PRRT treatments were performed by using [^177^Lu]Lu-DOTA-TATE (Lutathera^®^, Advanced Accelerator Applications, Saint-Genis-Pouilly, France). All subjects were referred to PRRT by a multidisciplinary team after an extensive workup, including a consultation by the nuclear medicine physician. If these and all other investigations and assessments were acceptable, patients’ preparation therapy with long- and short-acting cold somatostatin analogs were stopped at least 2 weeks or 24 h prior to treatment, respectively. On the day of treatment, as well as for the subsequent 2 days after, the patients received 8 mg of a serotonin-blocking agent to suppress nausea as well as a steroid. In order to prevent renal toxicity, an aminoacidic saline solution containing 25 g of lysine and 25 g of arginine was intravenously infused over four to six hours, starting 30 min before the administration of the radiopeptide. The administration of [^177^Lu]Lu-DOTA-TATE was performed over a period of approximately 30 min by a team of nurses, nuclear medicine physicians, and nuclear medicine technicians according to manufacturers’ recommendations at a fixed dose of 7.4 GBq per cycle [2,3]. No personalized dosimetry protocols were applied. A post-therapy whole-body scan (WBS) was performed 4, 48 h, and seven days after each treatment, using a SPECT/CT camera (NM680, Ge Healthcare, Chicago, IL, USA) for both diagnostic and dosimetric purposes to provide sufficient time points for time–activity curves. The imaging protocol included a WBS integrated using tomographic SPECT/CT acquisition to image the kidneys and the other organs at most risk; these images were acquired using an NaI (Tl) gamma camera equipped with medium energy collimators and a 15% energy window centered on the 208 keV photopeak. The X-ray CT data were acquired with automatic exposure control without intravenous contrast. The PRRT procedure was repeated four times for each patient at a time interval of eight weeks, according to the patient’s clinical condition, tumor load, and occurrence of any toxicities.

### 2.3. Renal and Bone Marrow Dosimetry

Dosimetry is mandatory to quantify and determine a threshold value of the adsorbed administered dose at the target lesions and the noble organs at risk (OAR), such as the bone marrow and the kidneys [12,13,14,15]. The evaluation of the absorbed administered dose can be obtained by converting the cumulative activity in the region of interest (ROI) into the absorbed dose (Gy). In order to obtain an accurate estimation of time–activity curves, several time points are required. In our department, our protocol includes three SPECT/CT acquisitions after each cycle, at 4, 48, and 168 h, respectively. The acquired images were reconstructed, corrected, and quantified by using an MIM application (SPECTRA Recon/Quant) and the images obtained at 48 h were considered references. The contouring of the ROI was obtained using a self-segmentation artificial intelligence (AI) algorithm (Contour Prontoge AI 1.1.2) and validated by a nuclear medicine specialist. The time activity curves (TACs) with bi-exponential fit from the volumes of interest (VOI) were acquired in the different time intervals, from which the residence time of the radiopharmaceutical was obtained both in the target lesions and in the OAR. According to the biodistribution of the tracer, the kidneys, bladder, liver, spleen, and bone marrow were considered as OAR. The dosimetric data were derived using the convolution method between the TAC coefficients and dose kernels [16,17]. For each patient, the software provides a dose volume, a mean dose (Gy), a minimum dose, and a maximum dose to both target lesions and OAR, VOI statistics, biokinetics of TACs, fits used, and residence time for all contoured ROIs.

### 2.4. Impact of PRRT on Renal and Bone Marrow Function

Before each cycle of treatment, as well as 3 months after the fourth cycle of PRRT, hematologic, liver, and kidney functions were monitored for each patient by the dosage of the following parameters: white blood cell (WBC) count, hemoglobin (Hb), platelets (PLT), aspartate transaminase (AST), alanine transaminase (ALT), creatinine, and estimated glomerular filtration rate (eGFR). The occurrence of toxicity was defined according to Common Toxicity Criteria for Adverse Events (CTCAE), Version 5 [18].

### 2.5. Follow-Up

All patients underwent laboratory and imaging procedures between January 2021 and December 2023. The response to therapy was considered as the endpoint. An objective tumor assessment on CT or MRI was performed 12 weeks after the date of the treatment according to RECIST criteria [19]. The patients were classified according to imaging results as having a complete response (CR), partial response (PR), stable disease (SD), or progression of disease (PD). Moreover, the response rate was calculated as the percentage of patients who had a CR or PR. Safety was assessed one month after the end of the treatment according to the occurrence of adverse events, based on the National Cancer Institute Common Terminology Criteria for Adverse Events (version 4.03). For this purpose, laboratory tests, physical examinations, vital signs, electrocardiography, ECOG, and Karnofsky performance status were considered.

### 2.6. Statistical Analysis

Continuous data were reported as mean ± standard deviation and categorical data were reported as a percentage. The differences in laboratories parameters among different time points groups were tested using repeated measures ANOVA followed by post hoc multiple comparisons with the Bonferroni correction. Statistical analysis was performed using Stata software version 20 (StataCorp LLC, College Station, TX, USA). A two-sided *p*-value < 0.05 was considered statistically significant.

## 3. Results

In the period from October 2020 to November 2023, 21 subjects were referred to [^177^Lu]Lu-DOTA-TATEPRRT, according to the regional multidisciplinary team. Baseline characteristics of the study population are reported in Table 1. A total of 17 (81%) patients were male, with a mean age of 65 ± 9 years. The more prevalent location of GEP-NETs was the small intestine (n = 12). All patients had metastases at diagnosis, and they were referred to PRRT for progressive disease. Only 1 patient had functional disease with uncontrolled carcinoid symptoms, including flushing, diarrhea, chronic nausea, and vomiting.

### 3.1. Renal and Bone Marrow Dosimetry

The mean adsorbed doses per administered activity were obtained for both OAR and target lesions. Specifically, the mean was 1.58 (1.34–1.90 Gy/GBq) for kidneys, 0.46 (0.15–0.85 Gy/GBq) for bladder, 2.76 (0.37–7.37 Gy/GBq) for liver, 1.20 (1.05–1.92 Gy/GBq) for spleen, and 0.23 (0.14–0.38 Gy/GBq) for bone marrow, with a body background of 0.50 (0.18–1.11 Gy/GBq). In addition, it was 6.78 (0.55–13.38 Gy/GBq) for hepatic metastases and 1.52 (0.29–3.85 Gy/GBq) for lymph nodes. Moreover, the mean time of radiopharmaceutical uptake was calculated in target lesions, and it was 12.1 h (0.03–113.6) for the liver and 5.3 (0.01–21.21) for lymph nodes.

### 3.2. Evaluation of Renal and Bone Marrow Function

The laboratory parameters for each cycle and 3 months after at the end of the treatment for all patients are reported in Table 2. As observed, no significant differences were observed among different controls in liver and renal functional parameters. However, there was a significant variation in both platelets and white blood cell counts among all-time points (both *p* < 0.05).

**Figure 1:**
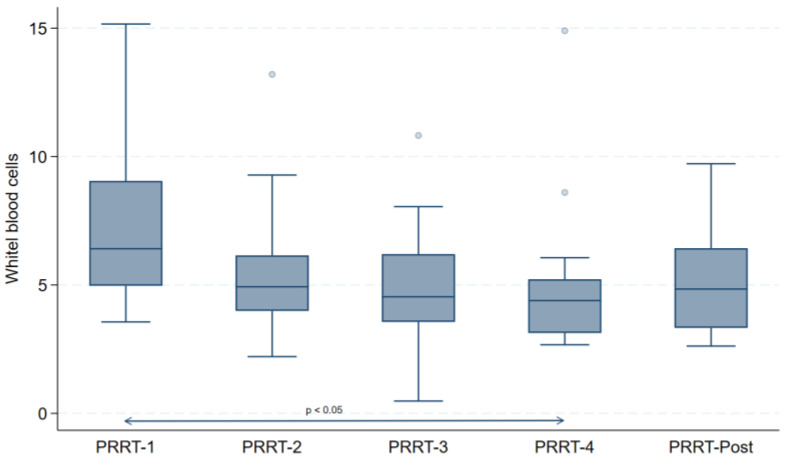

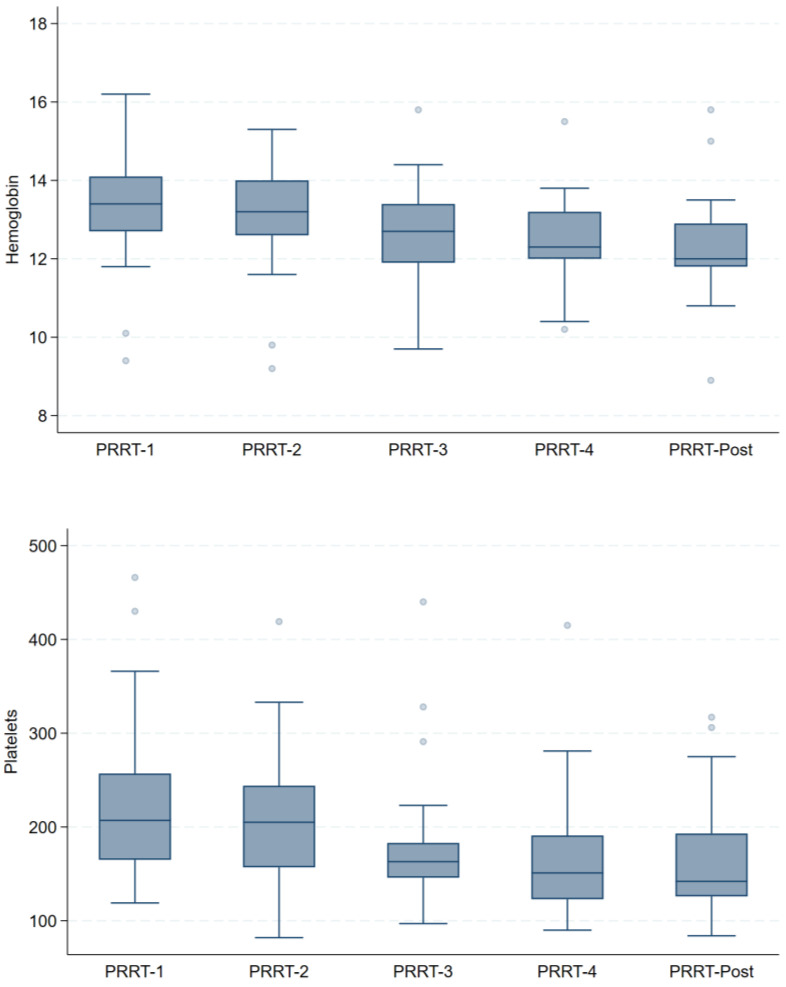
Comparison of white blood cells, hemoglobin, and platelets dosage before each PRRT cycle and 3 months after the treatment.

At post hoc analysis, only white blood cells showed a mild, albeit significant decrease between baseline values and the last treatment (*p* < 0.05) that disappeared 3 months after the end of the therapy (Figure 1).

### 3.3. Response to Therapy and Outcome

**Figure 2:**
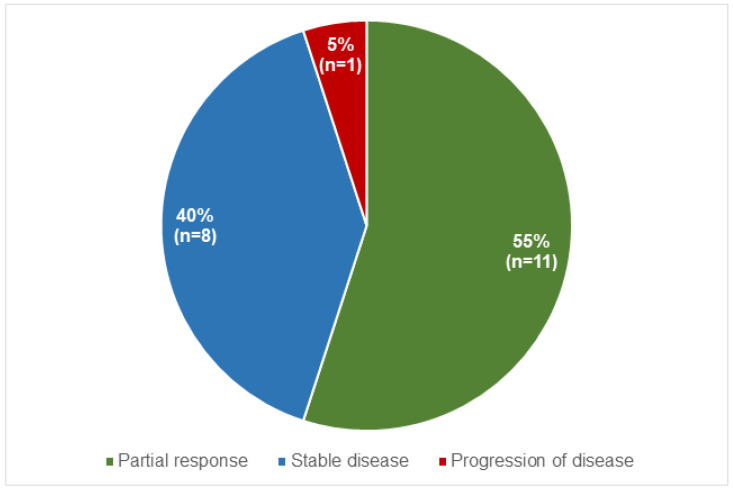
Distribution of response to therapy in the entire population according to RECIST criteria.

The response to therapy 3 months after treatment was evaluated in 20 (95%) patients. The response to therapy according to RECIST criteria is reported in Figure 2.

A total of 11 (55%) had a partial response, and 8 (40%) were found to have stable disease. Only one (5%) patient had progression of disease 4 months after treatment. Follow-up was completed for 18 (86%) patients. During a mean time of 22 ± 10 months (range 4–39), five (28%) had progression of disease, and one of these patients died 4 months after treatment. The remaining 13 (72%) patients were alive with stable disease at the end of follow-up.

**Figure 3:**
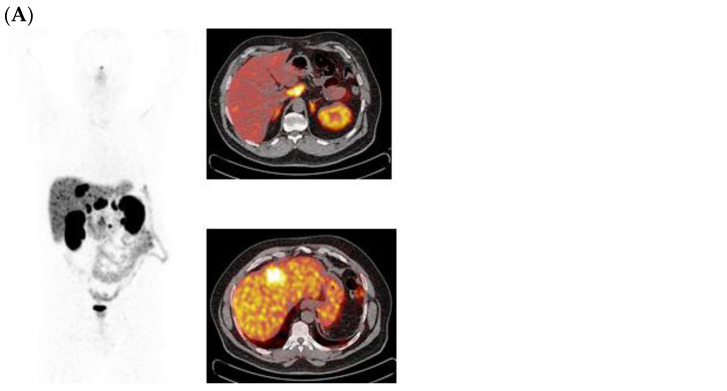

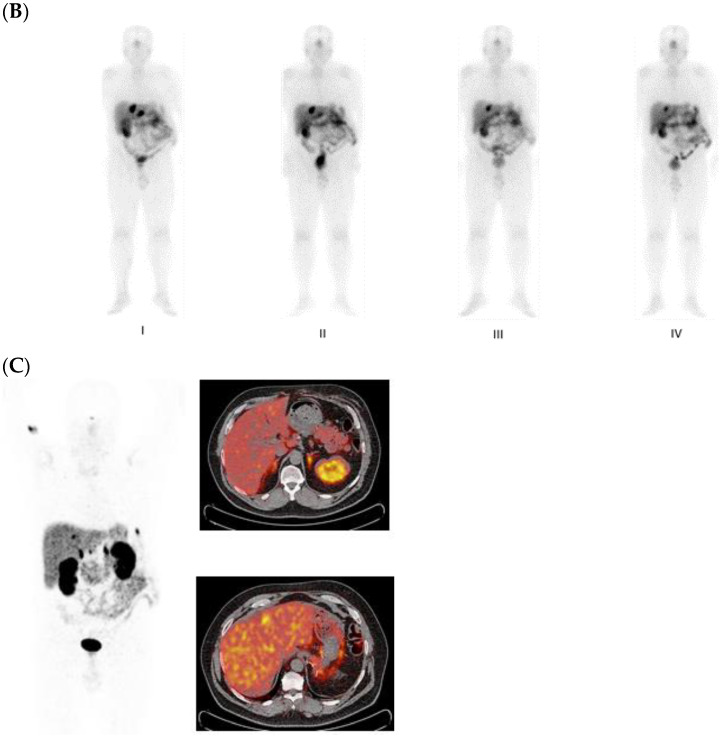
MIP views and transaxial fusion images of ^68^Ga-DOTATOC PET/CT scans performed before PRRT (**A**), WBS scans after [^177^Lu]Lu-DOTA-TATE (**B**), and MIP views and transaxial fusion images of a post-therapy ^68^Ga-DOTATOC PET/CT scan (**C**) in a patient with metastases to the liver and abdominal lymph nodes from pancreatic NET. The focal uptake on liver and lymph node metastases is reduced on the post-therapy images.

**Figure 4:**
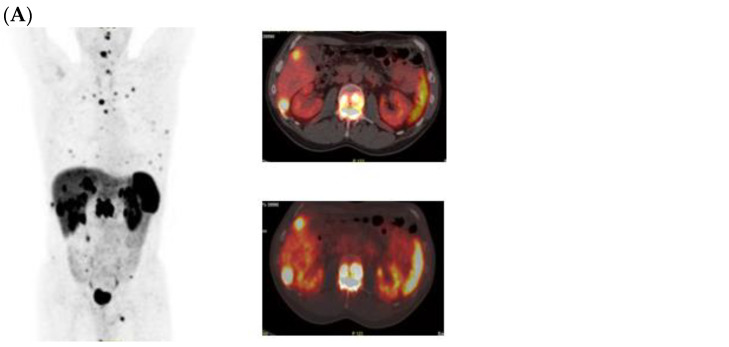

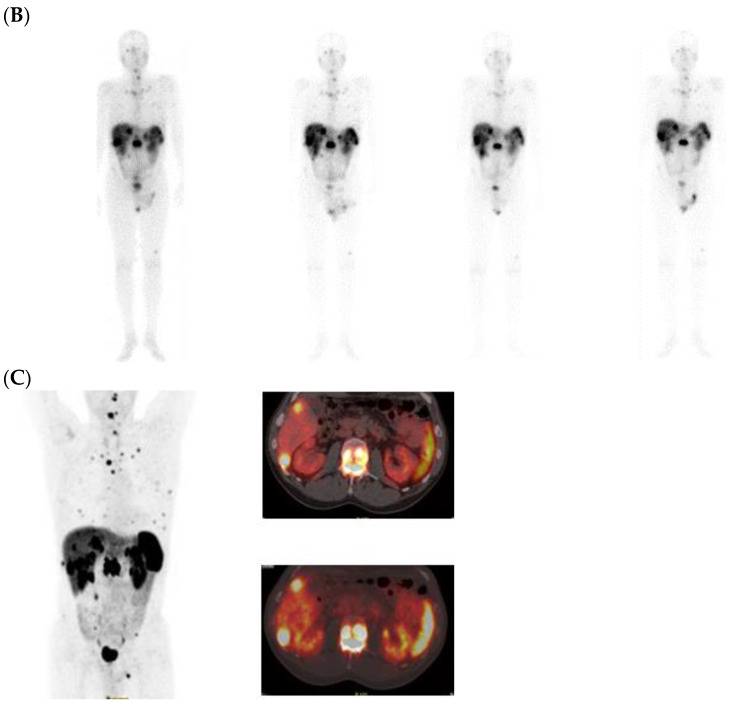
MIP views and transaxial fusion images of ^68^Ga-DOTATOC PET/CT scans performed before PRRT (**A**), WBS scans after [^177^Lu]Lu-DOTA-TATE (**B**), and MIP views and transaxial fusion images of a post-therapy ^68^Ga-DOTATOC PET/CT scan (**C**) in a patient with metastases to the bone, liver and abdominal lymph nodes from ileal G1 NET. The uptake on bone, liver and lymph node metastases is stable in the post-therapy images.

Two representative cases of GEP-NETs patients who underwent PRRT in our institution have been reported in Figure 3 and Figure 4.

## 4. Discussion

In this study, our single center experience in performing [^177^Lu]Lu-DOTA-TATE therapy in patients with GEP-NETs has been reported. We tested the safety and efficacy of PRRT in 21 patients treated in our institution according to the regional multidisciplinary team decision. Our data confirm that [^177^Lu]Lu-DOTA-TATE therapy is safe, and despite a mild and transient effect on bone marrow function, no significant adverse effects have been registered. Moreover, PRRT seems to be able to control the burden of disease [2,3]. 

The management of patients with GEP-NETs has significantly changed during the last decade. The overexpression of SSTR on the cell surface represents an ideal molecular target that can be bound by radiolabeled somatostatin analogs for both diagnostic and therapeutic purposes. The introduction of [^177^Lu]Lu-DOTA-TATE PRRT has significantly changed the management of GEP-NETs patients [1,2,3,4,5]. In patients with a significant expression of SSTR, assessed using PET/CT imaging with radiolabeled somatostatin analogs, the same compounds can be used labeled with lutetium-177 to obtain therapeutic effect, thanks to its ability to emit β-particles [9,20,21,22]. 

The [^177^Lu]Lu-DOTA-TATE has been approved as a therapeutic option for patients with progressive, advanced, well-differentiated G1 and G2 since the results of NETTER-1 were published [1,2]. 

Recently, the results of the NETTER-2 trial have been published [23]. In this recent report, both the efficacy and safety of [^177^Lu]Lu-DOTA-TATE were tested in 261 G2/G3 GEP-NETs patients with advanced grade 2–3, well-differentiated GEP-NETs from 45 centers across nine countries. In this important study, [^177^Lu]Lu-DOTA-TATE plus octreotide LAR significantly extended the median PFS in patients with G2/G3 GEP-NETs, compared to the control group (8.5 vs. 22.8 months). Therefore, [^177^Lu]Lu-DOTA-TATE should be considered as a first line of care in this type of patients.

After the use of PRRT increased worldwide, several institutions provided their experience testing the potential application of [^177^Lu]Lu-DOTA-TATE in real clinical practice [24,25,26,27,28,29].

Our department at the University Federico II of Naples takes part in the ENETS multidisciplinary network. As already mentioned, the main components of our approach include the following: establishing a NET multidisciplinary team, following these patients with a precise diagnostic and therapeutic path including different medical figures, and exploiting the potential of both morphological and molecular imaging methods in restaging and predicting response to therapy.

Since October 2020 to November 2023, 21 patients underwent [^177^Lu]Lu-DOTA-TATE therapy in our institution. Those patients have been addressed in PRRT according to the multidisciplinary team decision. Only patients with positive ^68^Ga-DOTATOC uptake, preserved bone marrow, and renal function were eligible to perform PRRT. In our population, PRRT was performed at fixed doses of 7400 MBq; however, dosimetry is routinely performed to monitor the adsorbed dose to both OAR and target lesions. According to European guidelines [30,31], dosimetry is mandatory for patients’ candidates to PRRT. However, the best approach has not been fully addressed yet despite the fact that it is essential in order to obtain a real individual and personalized approach for NET patients with metastatic disease [10,12,32]. In particular, for the evaluation of toxicity to OAR, 23 Gy and 2 Gy are considered the maximum safe dose to the kidney and to bone marrow, respectively [12]. Therefore, both the follow-up evaluation and planning the number of serial treatments could be based on post-therapy SPECT/CT, owing to the partial decay of ^177^Lu into photons. Ilan et al. [13] evaluated 24 patients with NETs treated with ^177^Lu-DOTATATE PRRT. The authors calculated the tumor-absorbed dose for metastatic lesions by using sequential SPECT/CT imaging. All studies were acquired 24, 96, and 168 h after PRRT infusion. Ilan et al. [13] observed a significant relationship between the absorbed dose and size reduction in tumor lesions, suggesting that metastases receiving higher absorbed doses are more likely to respond to PRRT [13]. In a study by Del Prete et al. [14], a similar result was shown by the renal absorbed dose after the first treatment by the body surface area and glomerular filtration rate. Moreover, for the subsequent cycles, renal dosimetry of the previous cycle was the only parameter able to predict renal absorbed dose. In addition, a lesion-absorbed dose >130 Gy was identified as a cut-off to obtain a reduction in tumor volume [14]. In our institution, the protocol is based on three WBS and SPECT/CT scan acquisitions in order to obtain enough time points. The results from our population show a mean adsorbed dose per administered activity for the target lesions of 6.78 (0.55–13.38 Gy/GBq), with acceptable values on OAR, in particular kidneys and bone marrow. 

The safety of [^177^Lu]Lu-DOTA-TATE has been previously evaluated [2,3,21,23]. The results from the NETTER-1 trial [2,3] showed a small number of grade 3 or 4 neutropenia, thrombocytopenia, and lymphopenia versus the absence of toxicity in the control group, highlighting the good tolerability of this treatment. Kwekkeboom et al. [21] confirmed that the PRRT was safe and well tolerated, with a low incidence of grade 3 or 4 hematologic toxicity (3.6% of administrations); only three patients showed myelodysplastic syndrome, and two patients had temporary liver toxicity. In our protocol, a mild and transient reduction in white blood cells and platelets (both *p* < 0.05) was observed among groups, without severe hematologic effects.

We also evaluated the efficacy of the treatment. In our population, in agreement with previous studies, most of the patients had partial response or stable disease 3 months after PRRT, according to RECIST criteria. Only one patient had progression of disease and died 4 months after the end of the therapy. Our data confirm that PRRT [^177^Lu]Lu-DOTA-TATE is safe and well tolerated, highlighting the need for an accurate selection of patients with preserved renal and bone marrow function.

This study has some limitations that must be considered. This is a retrospective study, where patients have been referred to PRRT according to standard indications. In addition, available follow-up time is relatively short and the study population is small. It should be considered that the sample size could have limited the statistical power of the test. In addition, in our analysis, a control group has not been considered. Therefore, a comparative analysis between patients treated with [^177^Lu]Lu-DOTA-TATE and patients who underwent other therapies has not been performed. Finally, due to the retrospective nature of this study, tumor markers performed during follow-up were not available for all the patients, despite that their prognostic implications in gastrointestinal oncology pathology, including GEP-NETs, have been extensively demonstrated [33,34]. These points may have limited the clinical value of our results.

## 5. Conclusions

This is single center experience on evaluating the safety and efficacy of [^177^Lu]Lu-DOTA-TATE therapy at the University Federico II of Naples. Our data confirm that [^177^Lu]Lu-DOTA-TATE therapy is safe and effective in controlling the burden of disease in patients with GEP-NETs.

## Figures and Tables

**Table 1:** Baseline characteristics of the 21 patients enrolled.

Characteristics	
Male gender, n (%)	17 (81)
Age at the time of therapy (years)	65 ± 9
Tumor location	
Small intestine, n (%)	12 (57)
Pancreas	8 (38)
Colon-rectum	1 (4)
Tumor grade	
Grade 1, n (%)	11 (52)
Grade 2, n (%)	10 (48)
Ki-67 index	6.1 ± 6.2
Site of metastases	
Lymphnodes, n (%)	11 (52)
Liver, n (%)	18 (86)
Bone, n (%)	5 (24)
Carcinosis, n (%)	2 (9)
Previous surgery, n (%)	8 (38)
Chemotherapy, n (%)	2 (9)
Interferon/sunitinib/everolimus, n (%)	5 (24)

**Table 2:** Laboratories parameters obtained before each PRRT cycle and 3 months after the treatment.

	PRRT-1	PRRT-2	PRRT-3	PRRT-4	PRRT-Post	*p* Value
Bone marrow reserve						
WBC (×10^3^/mL)	7.2 ± 2.9	5.4 ± 2.4	5 ± 2.2	4.9 ± 2.7	5.2 ± 2.23	<0.05
Hb (g/dL)	13.3 ± 1.6	13 ± 1.5	12.7 ± 1.4	11.8 ± 2.8	12.3 ± 1.4	0.09
PLT (×10^3^/μL)	235 ± 98	210 ± 76	188 ± 78	170 ± 73	167 ± 66	<0.05
Liver						
ALT (U/L)	20 ± 8	21 ± 7	25 ± 21	20 ± 11	22 ± 8	0.68
AST (U/L)	22 ± 7	23 ± 7	24 ± 11	23 ± 8	24 ± 6	0.96
Renal function						
eGFR (mL/min/1.73 m^2^)	75 ± 19	75 ± 18	76 ± 17	71 ± 18	73 ± 18	0.91
Creatinine (mg/dL)	1.03 ± 0.3	1.01 ± 0.21	1.01 ± 0.22	1.06 ± 0.25	1.01 ± 0.21	0.94

Values are expressed as mean value ± standard deviation. WBC, white blood cells; Hb, hemoglobin; PLT, platelets.

## Data Availability

The data presented in this study are available on request from the corresponding author. The data are not publicly available due to privacy restrictions.
